# Flavonoids and Their Anti-Diabetic Effects: Cellular Mechanisms and Effects to Improve Blood Sugar Levels

**DOI:** 10.3390/biom9090430

**Published:** 2019-09-01

**Authors:** Raghad Khalid AL-Ishaq, Mariam Abotaleb, Peter Kubatka, Karol Kajo, Dietrich Büsselberg

**Affiliations:** 1 Department of Physiology and Biophysics, Weill Cornell Medicine-Qatar, Education City, Qatar Foundation, Doha 24144, Qatar; 2 Department of Medical Biology and Department of Experimental Carcinogenesis, Division of Oncology, Biomedical Center Martin, Jessenius Faculty of Medicine, Comenius University in Bratislava, 03601 Martin, Slovak Republic; 3 Department of Pathology, St. Elizabeth Cancer Institute Hospital, 81250 Bratislava, Slovak Republic; 4 Biomedical Research Centre, Slovak Academy of Sciences, 81439 Bratislava, Slovak Republic

**Keywords:** diabetes mellitus, flavonoids, hyperglycemia, anti-diabetic, lipogenesis

## Abstract

Diabetes mellitus (DM) is a prevailing global health metabolic disorder, with an alarming incidence rate and a huge burden on health care providers. DM is characterized by the elevation of blood glucose due either to a defect in insulin synthesis, secretion, binding to receptor, or an increase of insulin resistance. The internal and external factors such as obesity, urbanizations, and genetic mutations could increase the risk of developing DM. Flavonoids are phenolic compounds existing as secondary metabolites in fruits and vegetables as well as fungi. Their structure consists of 15 carbon skeletons and two aromatic rings (A and B) connected by three carbon chains. Flavonoids are furtherly classified into 6 subclasses: flavonols, flavones, flavanones, isoflavones, flavanols, and anthocyanidins. Naturally occurring flavonoids possess anti-diabetic effects. As in vitro and animal model’s studies demonstrate, they have the ability to prevent diabetes and its complications. The aim of this review is to summarize the current knowledge addressing the antidiabetic effects of dietary flavonoids and their underlying molecular mechanisms on selected pathways: Glucose transporter, hepatic enzymes, tyrosine kinase inhibitor, AMPK, PPAR, and NF-κB. Flavonoids improve the pathogenesis of diabetes and its complications through the regulation of glucose metabolism, hepatic enzymes activities, and a lipid profile. Most studies illustrate a positive role of specific dietary flavonoids on diabetes, but the mechanisms of action and the side effects need more clarification. Overall, more research is needed to provide a better understanding of the mechanisms of diabetes treatment using flavonoids.

## 1. Diabetes and Flavonoids

### 1.1. Diabetes Mellitus

Diabetes mellitus (DM) is one of the epidemics challenging public health problems throughout the world [1]. The prevalence rate of diabetes is increasing exponentially and the World Health Organization predicts that by the year 2030, diabetes is expected to be the seventh leading cause of death worldwide [2,3]. Diabetes mellitus is a metabolic disorder characterized by the elevation of blood glucose due to the defects in insulin action, secretion or both (insulin is insufficient or inefficient) [4]. Type 1, type 2, and gestational diabetes are the three main types of diabetes targeting children, adults, and pregnant women, respectively [5]. The internal and external factors such as obesity, urbanization, genetic mutations, and a lack of physical activities contribute to the pathogenesis of diabetes [6]. The symptoms and signs of diabetes include polyuria (frequent urination), polyphagia (increased hunger), polydipsia (increased thirst), weight loss, and unconsciousness [7]. Diabetes could lead to deleterious complications like nephropathy, atherosclerosis, and cardiac dysfunction and target major organs in the body such as heart, nerves, kidneys, eyes, and blood vessels [8]. The high mortality and morbidity rate of diabetes combined with the higher risk of bacterial or viral infections or the development of cancer is a major concern of the diseases epidemic [9]. While currently there is no cure, diabetes is successfully treated by managing a healthy lifestyle combined with the administration of anti-diabetic agents and hypoglycemic drugs such as sulphonylureas, thiazolidinediones (TZDs), and biguanides all of which reduce blood glucose [10].

### 1.2. Glucose Homeostasis

Following a carbohydrate rich meal, the level of glucose in the body is regulated by two primary hormones: Insulin and glucagon [11]. The digestion of most starch molecules occurs in the upper gastrointestinal tract where they get hydrolyzed into smaller molecules (monosaccharides) which are absorbed through glucose transporters (GLUT) into the blood stream [12]. The GLUT-family is encoded by SLC2 genes and is responsible to transport monosaccharide, polysaccharide, and other small compounds through the membrane [13]. Fourteen GLUT proteins are expressed in human; GLUT 1-12, GLUT 14, and a myo-inositol transporter (HMIT) [14]. GLUT 2 is responsible to transport glucose from the circulation to pancreatic β cells where it gets oxidized and leads to the secretion of insulin [11]. The reduction of blood glucose levels occurs through three main mechanisms: (i) The enhancement of a glucose uptake by peripheral tissues through the translocation of GLUT 4; (ii) the inhibition of lipolysis and the promotion of lipogenesis; and (iii) the promotion of glucose storage and utilization in the liver [11]. One the other hand, when the glucose level in the body is low, the level of glucagon secretion increases due to two mechanisms: (i) The promotion of glucose production and release in the liver and (ii) the promotion of lipolysis and releasing free fatty acids from adipose tissue [14,15].

### 1.3. Insulin Resistance

Insulin resistance is defined as an impaired sensitivity to insulin due to an increase insulin secretion [16]. The different mechanisms explain the causes of insulin resistance in diabetic patients are: abnormal insulin production; impaired post-receptor signaling (major cause); insulin receptor mutation; and the presence of an insulin antagonist in the body [17]. The defect in glucose uptake due to the down-regulation of GLUT 4 translocation is considered to be the primary metabolic abnormality in type 2 diabetes which occurs as a result of tyrosine phosphorylation inhibition of insulin receptor substrate (IRS-1) [18]. The inhibition of an insulin signaling pathway occurs due to increased phosphatase activities, such as protein tyrosine phosphatase (PTP1B) and tensin homologue (PTEN), which dephosphorylate signaling molecules and inhibit insulin signal [19]. Cell exposure to free fatty acids (FFAs) and tumor necrosis factor alpha (TNF-α) inhibit the phosphorylation of IRS-1 that inhibit insulin signaling and action [20]. A suppressor of cytokine signaling (SOCS-1 and 3) shows a different underlying mechanism which blocks the downstream insulin signaling pathway by competing with IRS-1 to associate with the insulin receptor (IR) [21]. The accumulation of lipids in skeletal muscle and liver activates the pathways which negatively affect an insulin signaling pathway resulting in the reduction in both glucose uptake by skeletal muscle and glycogen synthesis in the liver [22]. In order to combat insulin resistance, the body increases the production of insulin to maintain euglycemia leading to an increase in the size of islet cells and pancreatic β-cells [23].

### 1.4. Insulin Release Defect in Diabetes

Type 2 diabetes is characterized by the high glucose level in the blood (hyperglycemia), the alteration in β-cells size and function, and insulin resistance [24]. Apoptosis caused by lipotoxicity, intracellular and extracellular deposit of islets amyloid polypeptide (IAPP), and glucotoxicity decreases the β-cells size which alter their functions [25]. As Β-cell mass decreases and its functions reduces, the cells are unable to compensate for the higher demand of insulin secretion due to insulin resistance [26]. Chronic hyperglycemia caused by chronic over-nutrition has proven to induce β cells apoptosis by endoplasmic reticulum stress (ER), a high level of intracellular calcium, the production of reactive oxygen species (ROS), and oxidation stress [27]. A high level of FFAs could also stimulate pro-apoptotic effects on β-cells that diminish their functions [28].

### 1.5. Lipogenesis Regulation in Adipocytes

Adipocyte differentiation, cellular lipid droplets accumulation, and adipogenesis occur in the body due to adipocyte transcription factors, like peroxisome proliferator-activated receptor gamma (PPAR γ) and adipokines, such as resistin [29]. PPAR γ is a nuclear hormone receptor which is expressed mainly in the adipose tissue [30]. It is important in glucose homeostasis, insulin sensitivity, adipogenesis, and energy metabolism. It also is critical in inducing the uptake of glucose and fatty acids by the cells [31].

Adiponectin, another regulating factor to lipogenesis, is primarily involved in improving glucose and lipid metabolism. It also inhibits the expression of multiple pro-inflammatory cytokines, such as tumor necrosis factor alpha (TNG-α), which promotes lipolysis and increases FFAs production [32].

The absence of leptin, an adipocyte-secreted hormone, leads to severe metabolic derangements. The main role of leptin is to regulate food intake and promote the oxidation of FFA in the peripheral tissues to prevent lipid deposition [33]. Insulin resistance leads to hyperleptinemia, which induces leptin resistance and results in lipotoxicity [34].

### 1.6. Diabetes Management

Diabetes is a lifelong multifactorial disease with micro- and macro-vascular complications. This has prompted different pharmacological and non-pharmacological therapeutic agents and measures to be implemented to benefit diabetic patients with the aim of enhancing their quality of life [35]. The currently available treatment for diabetes mainly manages to reduce and regulate glucose metabolism [36]. The first line of intervention for diabetic patients is to change their lifestyle to a healthier diet and to physical activities [37].

The administration of insulin is routinely used for type 1 diabetic patients as their pancreatic β- cells are incapable of secreting insulin, and type 2 diabetic patient’s due to their inability to respond to circulating insulin [38]. Managing diabetes is also achieved by using some antidiabetic compounds that reduce blood glucose levels. In addition, surgical operations, like bariatric surgery, can help obese patients with their diabetes management if other interventions become difficult to contain the disease and its complications [39].

Managing diabetes is not simple as it requires continuous support, medical attention, and education to patients to prevent serious complications. Sustainable management of diabetes is a global necessity due to the increase in the morbidity rate of the disease.

### 1.7. Impact of Diabetes on Selected Pathways

**Figure 1:**
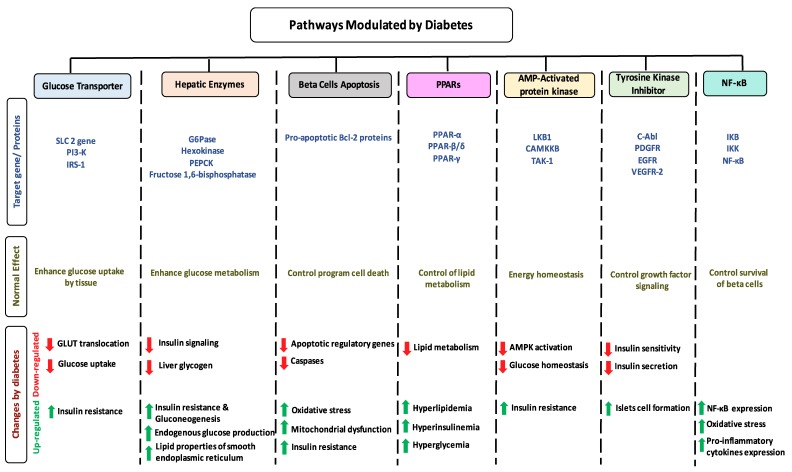
Schematic illustration of seven selected pathways modulated by diabetes. The figure is divided into seven columns and three rows. The column headings represent the pathways, while the rows heading represent: target genes/proteins for each pathway (blue), the overview physiological effect of these genes on pathways (Dark yellow), and changes occur on these pathways modulated by diabetes.

Micro and macrovascular complications of diabetes are associated with long term damage and organ failure [8]. A glucose transporter pathway is a rate limiting step for glucose utilization which is defective in diabetes [40]. Two isomers, GLUT1 and GLUT4, are involved in glucose transport through the cell membrane [14]. In diabetic patients, the translocation level of GLUT4 decreased which reduces glucose uptake and increases insulin resistance (Figure 1) [41]. The level and activity of hepatic enzymes, such as glucose-6-phosphate dehydrogenase, decreases with diabetes [42]. These changes enhance insulin resistance and gluconeogenesis and reduce insulin signaling and liver glycogen [43]. As a result of the reduced activity of peroxisome proliferator-activated receptor (PPARs) in diabetes, a significant reduction in lipid metabolism has been observed which leads to hyperinsulinemia and hyperglycemia [44]. Diabetes can also enhance the activation of apoptotic pathways by reducing the activity of apoptotic regulatory genes and caspases that upregulate mitochondrial dysfunction and insulin resistance (Figure 1) [45].

### 1.8. Dietary Flavonoids

Nutraceuticals are natural products derived from fruits and vegetables which provide multiple health benefits [46]. Scientific attention has been given over the past 20 years toward natural compounds, such as flavonoids serving as an antidiabetic agent [47].

Flavonoids are polyphenols which are ubiquitously found in daily consumed fruits, vegetables, nuts, cocoa, tea, grain seeds, and herbs [48]. They represent a large class of approximately 8000 phenolic compounds [49]. Flavonoids are considered as a class of biologically active secondary metabolites of plants known as pigment producers accountable for the odor and color of the flowers, where they serve antiviral, anti-allergic, antibacterial and anti-inflammatory functions [50]. The structure of flavonoids consists of 15 carbon skeletons and two aromatic rings (A and B) connected by a three-carbon chain which is usually an oxygenated heterocyclic C ring [51]. Based on the generic structure of a C ring, functional groups present on the ring, and the position where the B ring is attached to the C rings, six subclasses of flavonoids are defined: flavones; flavonols; flavanones; flavan-3-ols; isoflavones; and anthocyanosides [52].

Flavonoids have multiple positive health effects on metabolic disorders, such as cardiovascular disease, cancer, obesity, and diabetes [53]. Research and clinical studies have postulated the function of flavonoids in preventing and treating certain viral diseases like influenza [54]. They also serve as antioxidants which modulate oxidative stress in the body by neutralizing the effect of nitrogen and oxygen species, thus preventing the disease [55]. The antidiabetic activity of flavonoids supports the regulation of carbohydrate digestion, insulin signaling, insulin secretion, glucose uptake, and adipose deposition [56]. They target multiple molecules that are involved in the regulation of several pathways, like improving β-cell proliferation, promoting insulin secretion, reducing apoptosis, and improving hyperglycemia by regulating glucose metabolism in the liver [57]. A US study on 200,000 women and men evaluated the association between dietary intake of flavonoids subclasses and type 2 diabetes, confirming that a higher consumption of anthocyanins from apples, blueberries, and pears, lowers the risk of diabetes [58]. It is hypothesized that the majority of flavonoids bioactivity occurs due to their hydroxyl group, α, and β ketones [59].

### 1.9. Metabolism of Flavonoids

**Figure 2:**
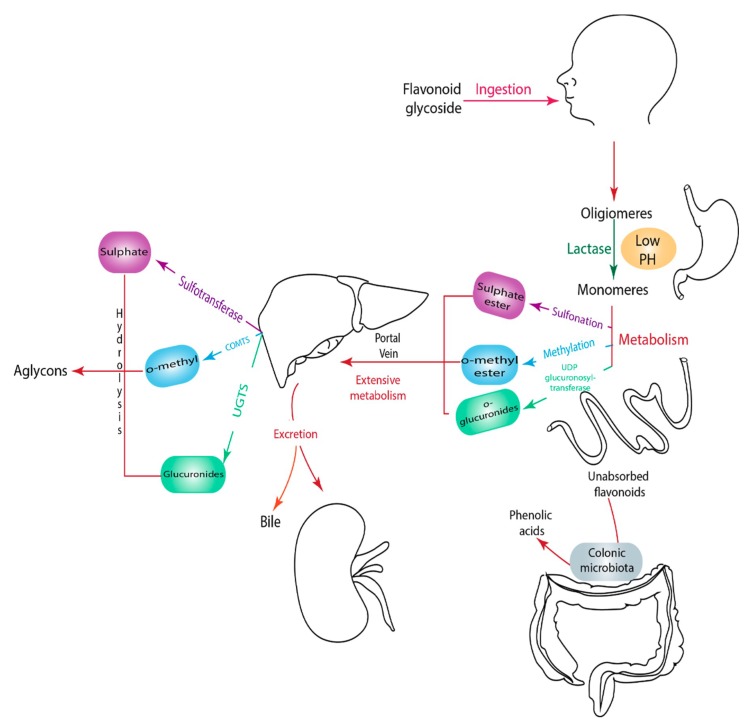
Illustration of a flavonoid pathway in the human body. The glycoside of flavonoids enters the body by an oral ingestion. An enzymatic reaction in the stomach (green arrow) breaks down the flavonoids to simpler molecules. In the small intestine, the first conjugation of flavonoids occurs where several reactions take place, such as sulfation and methylation, leading to the formation of *o*-glucuronides, *o*-methyl ester, and sulfate ester. The second conjugation of flavonoids take place in the liver to produce sulfates and glucuronides derivatives which could be excreted through bile and urine. Unabsorbed flavonoids enter the colon to be hydrolyzed or fermented into lower molecular compounds which can easily be absorbed.

Flavonoids hydrolyze and conjugate the main enzymes in the intestine, colon, and liver. In the intestine, the hydrolyzed and conjugated enzymes convert monomeric units of flavonoids into *O*-glucuronides, sulfate ester, and *O*-methyl ester [60]. The conjugation of flavonoids occurs in two phases: The small intestine (phase one), and then in the liver, the end of phase one and the beginning of phase two occurs. In the liver, the conjugated metabolites undergo further processing to produce sulfate and glucuronide derivatives where they get facilitated and excreted through bile and urine (Figure 2) [61]. Unabsorbed flavonoids move to the colon where they undergo hydrolysis or fermentation by colonic microbiota [62]. Flavonoids glucuronides in the liver are hydrolyzed by microbiota into aglycones where they break down further to lower molecular compounds that can be easily absorbed [63].

### 1.10. Search Strategy and Selection Criteria

Medline, Scopus, and PubMed were searched for papers published from 1985 using the search terms “flavonoids”, “phytochemicals”, “flavonoids AND anti-diabetics”, “flavonoids hyperglycemia”, “flavonoids lipid peroxidation AND diabetes”, “flavonoids AND diabetes mellitus”, “phytochemicals diabetes mellitus”, "flavonoids OR flavonoids subclasses AND diabetes”, and “flavonoids subclasses AND diabetes”. The search yielded approximately 5044 articles, and in this article, 240 articles were selected and analyzed in detail. 

## 2. Anti-Diabetic Effects of Selected Flavonoids

### 2.1. Flavonol

Flavonols are characterized by an unsaturated carbon ring at carbon 2–3 which is oxidized at C4 while hydroxylated at C3. They are found abundantly in lettuce, grapes, onions, kale, and berries [64].

#### 2.1.1. Quercetin

**Figure 3:**
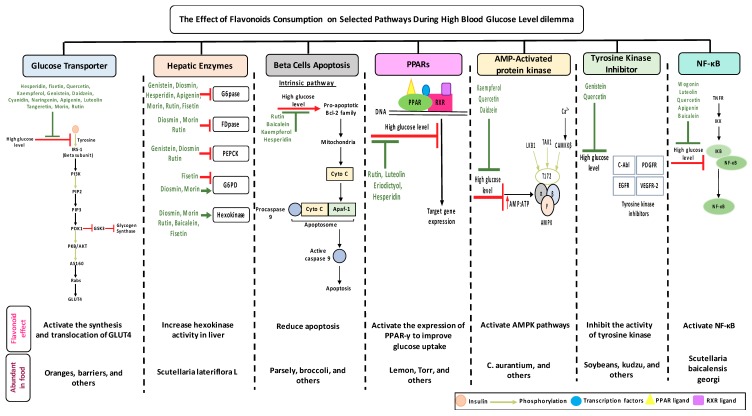
Flavonoids as anti-diabetic agents: Modes of Action. Aberrant signaling pathways (Glucose transporter, hepatic enzymes, beta cell apoptosis, PPARS, AMPK, Tyrosine kinase inhibitor, and NF-κB) and pathway components targeted by flavonoids (highlighted in green). Flavonoids have a wide range of anti-diabetic actions where one flavonoid could target multiple pathways. These phytochemicals can enhance or suppress (green and red lines respectively) the activity of GLUT 4 translocation, glucose uptake by the tissue, and hepatic enzymes activities; causes a decrease in apoptosis and tyrosine kinase inhibition that improves the pathogenesis of diabetes (see text for detailed modes) of action for flavonoids mentioned). For abbreviation, see abbreviation list.

3,5,7,3’,4’-Pentahydroxyflavone or quercetin dihydrate (C_15_H_10_O_7_) is the most abundant flavonoid in human dietary nutrition. It is found mostly in flowers, apples, seeds of tomatoes, berries, fennel, tea leaves, nuts, onions, broccoli, pepper, lovage, and shallots [65,66]. Quercetin acts as the base for the formation of other flavonoids skeletons, such as naringenin, rutin, and hesperidin [67]. Quercetin is involved in several biological actions such as: glucose homeostasis; insulin sensitizing and secreting; glucose utilization in peripheral tissues; the inhibition of intestinal glucose absorption [68]. Quercetin intake is inversely associated with the prevalence of T2DM in the Chinese population which suggests its preventive activity against T2DM [69,70]. A recent systematic review and meta-analysis of animal studies showed that quercetin decreases serum levels of glucose at doses of 10, 25, and 50 mg/kg of body weight [71]. Quercetin extracted from berries induced an insulin independent 5^’^ adenosine monophosphate-activated protein kinase (AMPK) pathway which slows the oxygen consumption of adenosine diphosphate by stimulating GLUT 4 translocation and expression in isolated mitochondria. This mechanism has a similar activity as metformin (medication used to treat type 2 diabetes) [72]. The antidiabetic action of quercetin involves the reduction of lipid peroxidation, glucose absorption by GLUT2, and the inhibition of insulin dependent activation of phosphoinositide 3-kinases (PI3K) [73,74]. In addition to this, quercetin and its derivatives (Table 1) stimulate a glucose uptake in muscle cells, and activate AMPK [75]. Treating streptozotocin (STZ)-induced diabetic rats with quercetin decreases the activity of glucokinase, hyperglycemia stimulating GLUT 4, hepatic gluconeogenesis, and glycogenolysis while it increases glucose liver uptake [76]. Quercetin supplementation for two weeks lowered the blood glucose level, upregulated the expression of genes involved in cell survival and proliferation in a liver, and enhanced the serum insulin in STZ- induced diabetic mice [77]. An injection of quercetin intraperitoneally into STZ- induced diabetic rats, reported a decrease in hyperglycemia, plasma cholesterol and triglycerides, and an improve glucose tolerance and hepatic glucokinase activity [78]. The co-treatment of quercetin and sitagliptin (a selective dipeptidyl peptidase-IV inhibitor) demonstrated an improvement in its oxidative and inflammatory status, metabolic profile, glycemic control, β-cells function, and islet structure in STZ- induced DM in rats [79]. Quercetin blocks the activities of a tyrosine kinase inhibitor, which has shown an effect against diabetes (Figure 3). The regulatory effect of quercetin to nuclear factor kappa-light-chain-enhancer of the activated B cells (NF-κB) also helps in improving glucose stimulated insulin secretion [80] (Figure 3).

#### 2.1.2. Rutin

Rutin is extracted from plants, such as oranges, lemons, grapes, peaches, limes, and buckwheat [81]. Rutin is also known as glycosylated quercetin, sophorin, and quercetin-3-*O*-rutinosie [82]. The anti-diabetic effects of rutin includes the reduction of carbohydrates absorption from the small intestine, the improvement of glucose uptake by tissues, the suppression of tissue gluconeogenesis, the activation of insulin secretion from β-cells, and the protection of the islets of Langerhans from degenerative changes. Rutin also lowers the formation of reactive oxygen species, advanced glycation end-product precursors, sorbitol, and pro-inflammatory cytokines [83]. Several experimental studies evaluated the hypolipemic and antihyperglycemic effects of rutin [84] (Table 1). The oral or intraperitoneal administration of rutin (50 mg/kg or 100 mg/kg) into a STZ model of type 1 diabetic rats significantly decreased glycated hemoglobin (HbA1c) and fasting blood glucose (FBG) [85]. When diabetic rats were fed with 100 mg/kg rutin, a significant increase in insulin levels and carbohydrate metabolic enzymes activity occurred. Further, the results showed a significant reduction in the level of plasma glucose [86]. The administration of rutin activates hepatic enzymes involved in the gluconeogenic and lipid metabolic process, such as hexokinase (Figure 3) [87]. The flavonoid also decreases significantly the level of urine protein, blood urea nitrogen, oxidative stress intensity, and fasting blood glucose. The treatment of rutin showed anti-apoptotic activities by increasing the activity level of B-cell lymphoma 2 (Bcl-2) and decreasing the level of caspase-3 in diabetic retina (Figure 3) [88]. Compared with other flavonoids like boswellic, quercetin, and ellagic acid, rutin was the most active flavonoid in reducing FBG, serum lipids, and improving glucose tolerance [89].

#### 2.1.3. Kaempferol

3,4,5,7-Tetrahydroxyflavone is a nontoxic flavonoid that is abundant in grapes, apples, onions, tomatoes, beans, kale, broccoli, potato, tea, and spinach [90]. Kaempferol has neuroprotective, antimicrobial, antioxidant, anti-inflammatory, and anticancer effects [91]. The extracts of kaempferol from *Bauhinia forficate* leaves reduce hyperglycemia and enhance glucose uptake, mimicking the action of insulin [92]. In vitro studies confirmed that treating with 10 μM of kaempferol enhances cellular viability and represses apoptosis [93]. Kaempferol has several antidiabetic effects, like improving AMP activated cellular protein expression and activation, reducing cellular apoptosis by suppressing caspase 3 activities, and increasing the production and secretion of insulin from β-cells [94] (Figure 3). In addition to this, kaempferol enhances glucose uptake by the cells through protein kinase C and PI3K pathways, and the synthesis of new glucose transporters [95]. The oral administration of kaempferol significantly decreased serum HbA1c levels, fasting blood glucose, and increased insulin resistance. This flavonoid decreased the genetic expression of PPARγ mediated through regulating AMPK activation [96] (Table 1). Moreover, kaempferol improved DM in STZ-induced mice through the promotion of glucose metabolism in skeletal muscle, and the suppression of hepatic gluconeogenesis [97]. In another study, kaempferol attenuated diabetic nephropathy in NRK-52E and RPTEC cells via suppressing RhoA/Rho-kinase mediated pro-inflammatory signaling (i.e., TNF-α, IL-1β, and TGF-β1) [98]. Like kaempferol, resveratrol is a natural phenol found in grapes, blueberries, and peanuts with potent antioxidant and anti-inflammatory activities that could effectively prevent diabetes [99,100].

#### 2.1.4. Isorhamnetin

An *O*-methylated bioactive compound is found commonly in medical plants, like *Oenanthe javanica* (Chinese celery, Japanese parsley, blume, minari in Korean), *Hippophae rhamnoides* (known also as sea- buckthorn), and *Ginkgo biloba* (commonly known as ginkgo) [101]. This flavonoid has anti-obesity and anti-diabetic effects [56]. The oral administration of isorhamnetin for 10 days into a streptozotocin**-**induced model of diabetes (STZ) at a dose of 10 mg/kg or 20 mg/kg showed an effective reduction in oxidative stress and hyperglycemia. The anti-diabetic effect of isorhamnetin is, not only limited to reducing the blood glucose level, but also it helps in reducing the accumulation of sorbitol level on rat lenses, the sciatic nerve, and red blood cells. [102]. An experimental study proposed that isorhamnetin glycoside has several effects on diabetes, like stimulating insulin secretion, the expression of enzymes involved in lipid metabolism, and the expression of endoplasmic reticulum stress markers [103,104].

#### 2.1.5. Fisetin

3,7,3’,4’-Tetrahydroxyflavoneis found abundantly in fruits and vegetables like apples, grapes, persimmon, cucumber, onions, and strawberries [105,106]. Fisetin possess anti-diabetic, anti-inflammatory, and neurotrophic effects [107]. The oral treatment of fisetin in a dose of 10 mg/kg for 30 days decreased Hb1Ac, blood glucose levels, and the expression of the gluconeogenic genes protein level, while it increased the concentration of plasma insulin [108]. In an in vivo study, the results showed that treatment with fisetin significantly reduced the level of NF-κB p65, Hemoglobin A1C (HbA1c), serum nitic oxide (NO), and blood glucose [109]. Fisetin also inhibits high glucose induced cytokine production in monocytes which could prevent diabetes [110]. The anti-diabetic effects of fisetin on hepatic enzymes include enhancing the activities of hexokinase, while reducing the activities of glucose 6 phosphate dehydrogenase (G6PD) and glucose 6-phosphatase (G6Pase) (Figure 3). Moreover, fisetin improves glucose homeostasis by attenuating carbohydrate metabolism enzymes in STZ diabetic rats [111]. Fisetin has been reported to improve the development of diabetic cardiomyopathy in STZ- induced DM rats by improving hyperglycemia/hyperlipidemia-mediated oxidative stress, the inflammation processes, and the programmed cell death [112]. Preclinical evidence illustrated the therapeutic potential of fisetin in diabetic neuropathy through the modulation of NF-κB and Nrf2 signaling pathways [113].

#### 2.1.6. Morin

A natural flavonoid, morin, is found mostly in traditional medical herbs, like *Prunus dulcis, Chlorophora tinctoria* L., and fruits such as guava and figs [114]. The oral administration of morin for 30 days in animal models resulted a significant improvement in glucose tolerance, hyperglycemia, and insulin resistance [115]. Diabetic rats were reported to have declined lipid peroxides and antioxidant levels after the treatment with morin [116]. Morin effectively decreased the level of inflammatory cytokines, like IL-6 and TNF-α, which proves its anti-inflammatory effects [117]. In animal models, morin recovered leptin sensitivity and hepatic insulin led to the reduction of liver lipid accumulation and hyperlipidemia [118]. Morin has different effects on hepatic enzymes where it is significantly reduces the activity of G6Pase and Fructose-1,6-diphosphatase (FDPase), while enhancing the activity of hexokinase and G6PD [119] (Figure 3).

### 2.2. Flavanones

Flavanones are known as di-hydroflavones and they are characterized by an oxidized, saturated carbon ring. Flavanones are widespread in citrus fruits and known for their free radical scavenging ability and antioxidant activity [64].

#### 2.2.1. Hesperidin

5,7,3’-Trihydroxy-4’-methoxyflavanone, a saturated oxidized aglycon, is found abundantly in citrus fruits, such as limes and lemons, tomatoes and cherries [120]. The effects of hesperidin and its glycoside (Table 2) are not limited to diabetes, but also have vascular, neuroprotective, anti-allergic, anti-inflammatory, anticarcinogenic, and antioxidant effects [121]. A study in db/db C56BL6 mice showed that hesperidin supplementation to the regular diet helps in regulating the activity of gluconeogenesis and glycolytic hepatic enzymes, and in improving hyperglycemia [122]. In db/db mice, the flavonoid has a very effective machinery, like increasing triglyceride fecal excretion and inhibiting lipid metabolizing enzymes which enhances the lipid metabolism activities [123]. Hesperidin effectively lowers blood glucose levels by upregulating GLUT 4 translocation and PPARγ [124]. Hesperidin supplementation showed a decrease in glucose 6 phosphatase (G6Pase) activities in STZ- induced diabetic rats, which diminish glucose exports from the cells by a glucose transporter membrane protein [125] (Figure 3). A dose of 10 g/kg diet of hesperidin treatment decreases glucose levels by altering glucose regulating enzyme activities [126]. The administration of hesperidin and hesperetin together have different effects on lipid and glucose metabolism and show lipid lowering activities [56]. Hesperidin also positively regulates the α-Klotho/FGF-23 pathway in STZ- induced DM rats, which demonstrate positive effects on diabetic toxicity in the liver and kidney [127].

#### 2.2.2. Naringenin

5,7,4’-Trihydroxyflavanone, a saturated oxidized aglycone, is present abundantly in citrus fruits, such as oranges, tomatoes, and grapefruits [128]. It has a wide range of biological activities, such as antioxidant, antidiabetic, anti-inflammatory, anticancer, and anti-mutagenic abilities [129]. Both naringenin and its glycoside (Table 2) possess anti-diabetic and anti-obesity activities [130]. An in vitro study presented that naringenin mimics the effect of insulin by decreasing pro-liprotein B secretion in liver cells [131]. The administration of this flavonoid (25 mg/kg) into diabetic rats showed a significant inhibition of α-glucosidase activity which delayed carbohydrate absorption, therefore, reducing postprandial blood glucose levels [132]. In type 2 diabetic mice, naringin could upregulate GLUT4, and regulates the expression of hepatic enzymes involved in gluconeogenesis and glycolysis which improves hyperglycemia [133,134]. In an everted rat intestinal sleeve, naringenin was found to inhibit the uptake of glucose by inhibiting sodium-glucose co-transporters in the intestine [135]. The administration of naringenin presented various effects in different diabetic rat models: (i) In STZ- induced diabetic rats, the flavonoid decreased the level of plasma glucose; (ii) in fructose-fed insulin resistance rats, naringenin improved insulin sensitivity; in HFD mice, it helped in the reduction of insulin resistance [136,137,138]. Treating diabetic mice with 25 mg/kg for 45 days significantly reduced hyperinsulinemia, hyperglycemia, decreased lipid membrane peroxidation, improved hepatic markers, restored the changes in lipid profile, and enhanced the antioxidants activities [139]. Naringenin anti-diabetic effects in diabetic rats were characterized by anti-oxidant, and anti-apoptotic activities which showed the potential of naringenin to limit neurodegeneration and prevent retinal damage in diabetic retinopathy [140].

#### 2.2.3. Eriodictyol

Eriodictyol, present in lemon fruits, significantly controls obesity and diabetes [141]. Recently, eriodictyol was identified as a novel insulin secretagogue in vitro and in vivo which exerts an exclusive glucose-dependent insulinotropic activity via a cAMP/PKA pathway [142]. Moreover, in diabetic rats, eriodictyol supplementation can effectively suppress oxidative stress [143]. The treatment with eriodictyol upregulated the expression of PPARγ2 and the adipocyte-specific fatty acid binding protein [144]. Furthermore, eriodictyol treatment significantly suppressed diabetes related lipid peroxidation [145] (Figure 3). Recently, eriodictyol was described as a protector of the rat retinal ganglial cells (RGC)-5 from high glucose-induced oxidative stress, inflammation, and cell apoptosis via the activation of Nrf2/HO-1 signaling [146].

### 2.3. Flavones

The structure of flavones is comprised of an unsaturated carbon ring at C2–3 and a ketone group at C4, but they lack the hydroxylation at carbon 3 if compared to flavonols. They are widely synthesized in flowers, leaves, and fruits [64].

#### 2.3.1. Apigenin

5,7,4’-Trihydroxyflavone is a phytoestrogen aglycoge found mostly in nuts, vine spinach, oranges, celery, garlic, tea, oregano, carrot, and chamomile [147]. In alloxan induced insulin dependent diabetic mice, the oral administration of apigenin for 10 days reduced hepatic antioxidants, like catalase, glutathione, and superoxide dismutase. The treatment with apigenin helps in reducing hyperglycemia, serum cholesterol and G6Pase activities in the liver (Figure 3) [148]. In STZ- induced diabetic rats, the administration of apigenin (4 mg/kg) showed a significant anti-hyperglycemic effect [149]. Apigenin treatment could prevent induced apoptosis through the inhibition of NF-κB activation [150]. In HepG2 hepatocytes, apigenin enhances the phosphorylation of AMPK. This effect of apigenin is 200 times more potent than metformin [49]. The enhancement of GLUT4 translocation upon the treatment with apigenin suggested its effect on lowering blood glucose [151]. Apigenin administration improved oxidative damage of pancreatic β-cells in STZ-induced rats by lowering cellular DNA damage, ROS production, protein carboxylation, lipid peroxidation, and restored cell apoptosis [152]. The administration of apigenin improved STZ-induced diabetic nephropathy through MAPK-NF-κB-TNF-α and TGF-β1-MAPK-fibronectin signaling [153].

#### 2.3.2. Luteolin

5,7,3’,4’-Tetrahydroxyflavone is a well-known anti-inflammatory and antidiabetic agent [154]. It is abundantly found in fruits and vegetables like cabbage, onion leaves, celery, carrots, parsley, peppers, broccoli, and apple skin [155]. Luteolin was reported to initiate insulin action and to enhance the expression of PPARγ target genes (Figure 3) in primary mouse adipose cells [156]. In damaged pancreatic cells, β cells, this flavonoid improves insulin secretion in uric acid by decreasing micro-autologous fat transplantation (Maft), a trans activator of insulin gene through NF-κB signaling pathway [157]. The anti-diabetic effects of luteolin (LU) and luteolin-7-*O*-glucoside (LUG) improve blood glucose, HbA1c, insulin, and HOMA-IR levels, and inhibit lipid synthesis [158]. Luteolin improves insulin resistance and adipose tissue inflammation by altering M1-like macrophage polarization in adipose tissue [159].

#### 2.3.3. Tangeretin

5,6,7,8 4’-Pentamethoxyflavone is a flavonoid prevalent in citrus fruits, such as oranges, citrus peel of tangerine, and mandarins [160]. The administration of tangeretin (200 mg/kg) in HFD-induced obese mice reduced blood glucose, total cholesterol, body weight and regulated adipocytokines, like leptin, IL-6, and adiponectin [161]. Treating diabetic rats with tangeretin (100 mg/kg) for 30 days reduced glucose plasma levels, Hb1Ac, and enhanced glycolytic enzymes, the level of insulin and hemoglobin significantly [162]. In 3T3-L1 preadipocyte, tangeretin increases the secretion of the insulin sensitizing factor while decreasing the secretion of the insulin resistance factor [163]. In addition, tangeretin down-regulates STZ-induced programmed cell death in INS-1 cells through the regulation of NF-κB signaling [164].

#### 2.3.4. Chrysin

5,7-Dihydroxyflavone is found abundantly in honey, fruits, bee pollen, propolis, and medical plants, such as *Passiflora caerulea* L. and *Tilia tomentosa* [165]. This flavonoid is an analog to apigenin but with lower bioavailability due to rapid excretion and metabolism [166]. Chrysin treatment in STZ-induced rats reported an elevation of glucose, MDA, TG, TC, LDL-C and a reduction of HDL-C, total protein, SOD, CAT, and GST [167]. The treatment with chrysin demonstrated an improvement in renal pathology and suppressed collagen-IV protein expressions in renal tissue [168]. In HFD/STZ-induced diabetic rats, chrysin significantly prevented the development of diabetic neuropathy (DN) due to the reduced level of pro-inflammatory cytokines in the serum [169]. Chrysin treatment decreases lipid peroxidation, glucose levels and increases insulin levels in diabetic rats [170]. The data suggest that chrysin has anti-diabetic and antihypertensive effects [171].

#### 2.3.5. Wogonin

5,7-Dihydroxy-8-methoxyflavone is a flavonoid extracted from the root of *Scutellaria baicalensis* and it has been used as a traditional medicine in East Asian countries [172]. It is associated with anti-inflammatory, neuroprotective, anti-viral, anti-bacterial, and antioxidants effects [173]. Wogonin has several beneficial effects on insulin sensitivity, blood glucose, and lipid metabolism through the activation of AMPK and PPARα [174]. The pretreatment with wogonin attenuated vascular inflammatory effects seen in high glucose induced (HG) vascular inflammation [175]. A preclinical study found that the anti-oxidative and anti-inflammatory of wogonin could attenuate diabetic cardiomyopathy [176].

#### 2.3.6. Diosmin

A naturally occurring flavonoid glycoside was first isolated from *Scrophularia nodosa* L. in 1925. It can be isolated either from several plant sources or by the dehydrogenation of the flavanone glycoside, hesperidin [177]. The diosmin administration to type 1 diabetic patients showed a decrease in HbA1c and an increase in glutathione peroxidase (GPx) (Table 3) [178]. Rats treated orally with diosmin for 45 days showed a significant decrease in plasma glucose levels, G6Pase, FDPase and an increase in G6PD and hexokinase (Figure 3) [179]. The diosmin treatment at a dose of 50 and 100 mg/kg for one month improved oxidative stress and hyperglycemia in diabetic rats [180]. Diosmin activates imidazoline receptors which increase adrenal β-endorphin secretion and, thereby improving metabolic homeostasis and the alleviation of serum glucose and lipids in STZ-induced type 1 diabetic rats [181].

#### 2.3.7. Baicalein

5,6,7-Dihydroxyflavone aglycone is isolated from the roots of *Scutellaria baicalensis* and fruits of *Oroxylum indicum* L. [182]. It exhibits extensive ant-inflammatory, anti-neuro-degenerative, and anti-cardiovascular effects [183]. The administration of 0.25 g or 0.5 g of baicalein to high fat diet (HFD) induced mice displayed a significant improvement in glucose tolerance, insulin levels, and hyperglycemia [184]. Diabetic rats treated with baicalein showed a substantial decrease in fasting blood glucose levels, HbA1c, food intake, and body weight [185]. Moreover, the treatment with baicalein was reported to decrease the TNF levels, advanced glycation end-products (AGEs), and NF-κB activation (Figure 3) [186]. The baicalein mechanism of action could upregulate AMPK signaling pathways, which can attenuate insulin resistance and inhibit inflammation [187]. The treatment of baicalein in HepG2 cells (0.001 umol/L and 0.01 umol/L) enhanced glucose uptake and glycolysis and suppressed [188]. In addition, baicalein prevents oxidative stress and inflammation in diabetic cardiomyopathy rats through the regulation of PI3K/AKT signaling [189]. Baicalein alleviates hepatic inflammation in diabetic db/db mice via the modulation of HMGB1/TLR4/NF-κB signaling [190].

### 2.4. Isoflavones

Isoflavones are found mostly in legumes, soybeans, and some microbes [64]. Genistein and daidzein are the major source of isoflavones. They have shown to have an anti-diabetic effect by stimulating insulin secretion from the pancreatic beta cells [56].

#### 2.4.1. Genistein

5,7,4’-Trihysroxyisoflavone, a naturally occurring soy isoflavone, is present numerously in soy, soybean products, and Chinese plants [191]. Genistein exerts the anti-diabetic effects by enhancing plasma lipids [192]. Genistein supplementation in type 1 diabetes animals led to the improvement of insulin levels and glucose metabolism [193]. An in vivo study found that genistein improved hyperglycemia through promoting cAMP/PKA signaling pathways [194]. The administration of genistein to rats fed with a fructose rich diet showed a protective role on renal malfunction through the modulation of insulin resistance [195]. The supplementation of genistein (0.02% in diet) in non-obese diabetic (NOD) rats showed the onset of diabetes was prevented and glucose homeostasis was improved through the preservation of β cell functions [196]. The beneficial effects were observed in non-generic mouse models ingested with 250 mg/kg of genistein like reduction in the fasting glucose level and β cell mass [197]. In STZ-induced mice, genistein improved glucose tolerance, hyperglycemia, and the level of circulating insulin [198]. Genistein demonstrated an inhibitory effect on tyrosine kinase which dysregulates glucose homeostasis (Figure 3) [199]. The administration of genistein to mice reduced body weight and improved glucose and lipid metabolism [200]. A transcriptome analysis revealed that genistein could affect the regulation of the hypothalamic circadian rhythms which could provide a novel target for the therapy of diabetes and obesity. Moreover, genistein has a protective effect against inflammation, neuropathic pain, and oxidative stress [201].

#### 2.4.2. Daidzein

7,4’-Dihydroxyisoflavone is a phytoestrogen mainly isolated from nuts, fruits, and soybeans [202]. Daidzein exerts an anti-diabetic effect by enhancing lipid and glucose metabolism [203]. Daidzein has promising therapeutic potential on impaired glucose, lipid metabolism, and vascular inflammation associated with T2DM [204]. Moreover, daidzein treatment in gastrocnemius muscle is effective in decreasing blood glucose, total cholesterol, and AMPK phosphorylation (Figure 3) [201]. Pure synthetic daidzein administered to hamsters significantly lowered plasma total cholesterol levels and blood glucose compared to the control group [205].

### 2.5. Anthocyanins

A water soluble, unoxidized, unsaturated flavonoid, anthocyanin, is present abundantly in flowers and fruits. The dietary consumption of this flavonoid is higher compared to other flavonoids. Several studies, both in animal models and cell lines, suggested that anthocyanins have anti-diabetic activities [56].

#### 2.5.1. Cyanidin

A flavonoid commonly distributed in vegetables, fruits, crops, and other plant-based diets [206], cyanidin, exerts an anti-diabetic effect by inhibiting intestinal α-glucosidase and pancreatic α-amylase [207]. In STZ-induced diabetic rats, cyanidin reversed degenerative changes in β-cells by preventing pancreatic apoptosis and activating insulin receptor phosphorylation [208]. Cyanidin-3-glucoside (C3G), a prominent anthocyanidins in the diet, improved antioxidant status and protected hepatocyte from oxidative stress against high glucose induced damage (HG) (Table 4) [209].

#### 2.5.2. Delphinidin

A flavonoid, delphinidin, is profusely found in pigmented vegetables and fruits, like berries, sweet potato, red cabbage, tomato, eggplant, carrots, red onion, and grapes [210]. It possesses anti-inflammatory, antioxidant, anti-mutagenic, and anti-angiogenic activities [211]. In an in vivo study, delphinidin showed to prevent endothelial cell function injuries associated with diabetes [212]. The administration of 100 mg/kg delphinidin to diabetic mice showed a decrease in HbA1c glycation and the rate of albumin [213]. Delphinidin and cyanidin were reported to reduce inflammation and regulate redox signaling pathways by ameliorating insulin resistance in high fat-fed mice [214]. The antidiabetic effects of delphinidin are due to their ability to reduce glucose uptake in mice jejunal tissue and human intestinal cells lines through free fatty acid receptor 1 (also named GPR40) [215].

#### 2.5.3. Pelargonidin

A flavonoid, pelargonidin, is abundantly found in berries, like blueberries, cranberries, and raspberries [216]. The treatment with pelargonidin helps reduce hyperglycemia and oxidative stress levels [217]. In diabetic rats, pelargonidin showed it reduces the formation of thiobarbituric acid reactive substances (TBARS) and antioxidant defensive enzymes levels [218]. In an in vitro study, pelargonidin and its aglycone, pelargonidin-3-galactoside, stimulated insulin secretion [219].

## 3. Challenges Using Flavonoids

Flavonoids have been proven to be strong candidates to reduce the pathogenesis of diabetes and its complications. The modulatory anti-diabetic effects of flavonoids reduce apoptosis and insulin resistance and enhance insulin secretion and GLUT 4 translocation.

### 3.1. Estimated Consumption Level of Flavonoids

Flavonoids derived from vegetables and fruits are consumed in low quantities. Moreover, the content of vegetables and fruits contain not only flavonoids, but also a mixture of secondary plant metabolites. Therefore, it is difficult to stimulate this mixture into a simple purified dietary supplement [220,221]. Efforts have been made to establish an optimal human dietary consumption level of flavonoids worldwide, but the estimate methods used were poorly established [222]. A U.S. study on 805 men aged 65–84 years reported that the estimate intake of flavonoids from quercetin, myricetin, kaempferol, apigenin, and leuteolin was 26 mg/d and the major sources of intake were in apples, tea, and onions [223]. Another study conducted in the Netherlands reported a two-times higher the level of flavonoids consumed in adults compared to the U.S. study (50 g/day) [224]. In addition, two Dutch studies reported the estimated consumption level of flavonoids to be 23 mg/day and 26 mg/day respectively [225,226]. These differences observed in the consumption level of flavonoids depend on dietary habits, geographical location, socioeconomic status, food processing and preparation method, solubility of flavonoids, and the ethnicity of the population. For example, in Japan, soy containing food is highly consumed and, as a result the intake of isoflavone is higher than other flavonoids subclasses [106]. A study reported that orange juice contains 81–200mg/L of soluble flavanones compared to 206–644 mg/L seen in the cloud which clearly suggest that processing and storage affects the concentration of flavonoids [227].

To date, no recommended dosage of flavonoids has been reported due to the heterogeneity of their molecular structure and the limited information about their bioavailability. Major advances in understanding flavonoids bioavailability have been made, but the challenge to overcome problems, such as cellular permeability, solubility, excretion, and metabolic alternation, are still lacking. Research groups are trying to enhance flavonoids bioavailability by targeting absorption sites, improving metabolic stability and intestinal absorption [50].

#### 3.1.1. Possible Side Effects of Flavonoids Consumption

Flavonoids in bacterial and mammalian experimental studies using Ames test indicated possible genotoxicity and mutagenicity of flavonoids if consumed at higher concentrations (ranges from 12.1 nmol to 225.0 nmol) [228]. Furthermore, it may alter amino acid, drug metabolism and the activity of key metabolizing enzymes [229]. Quercetin, a predominant flavonol in the human diet, showed a mutagenic effect by altering base-pair substitution and frame-shift mutation [230]. The isolated nuclei from liver rats treated with morin and naringenin showed an increase in reactive oxygen species, like hydroxyl radicals that lead to DNA degradation [231]. Additionally, flavonoids exert a cytotoxic activity as a topoisomerase II inhibitor. Genistein and quercetin are identified as topoisomerase II inhibitors, even at low concentrations (10 μM), where they accumulate cleavable complexes seen in patients with secondary leukemia [232]. Genistein, naringenin, kaempferol, and daidzein were reported to inhibit thyroxine synthesis by irreversibly inhibiting thyroid peroxidation [233].

Although no data are available to state the long-term side effects of increased flavonoid consumption, following an Asian diet that contains 68 mg of flavonol and 20–240 mg of isoflavone could improve thyroid function, reduce breast cancer mortality, and should not cause adverse health effects [234].

The concentrations needed for most flavonoids to generate mutagenic and cytotoxic side effects are unlikely to occur through dietary sources, but with supplementation, it could result in an increased toxic level. For instance, the recommended dosage of quercetin supplements is between 500 mg/day and 1000 mg/day, which is 20 times higher with what could be consumed in a vegetarian diet [235].

#### 3.1.2. Could Flavonoid Combinations have synergistic effects?

While the amounts of flavonoids consumed is crucial to establish positive effects but also to avoid negative effects, the tables list some flavonoids that trigger multiple selected pathways improving the pathogenesis of diabetes (Figure 3, Table 1, Table 2, Table 3 and Table 4). The better activity can be defined by the number of diabetes related pathways which are improved through the consumption of different flavonoids. The administration of baicalein triggers four pathways: The suppression in the NF-κB pathway and fatty acid synthesis; the activation in hexokinase activity in the liver; and the protection against cell apoptosis. Quercetin prompts the activity of three different pathways: It improves GLUT 4 translocation; inhibits tyrosine kinase activity; and reduces lipid peroxidation. β-cells apoptosis could be prevented by the administration of cyanidin or kaempferol, or baicalein. The consumption of rutin or cyanidin inhibits α-glucosidase and α-amylase which reduce carbohydrate absorption in the small intestine (Table 4).

Could their positive effects on diabetes be further improved by ingesting a combination of different flavonoids which complement each other by triggering additional pathways? For example, the administration of baicalein and quercetin initiates the positive effects on diabetes in six major pathways: The glucose transporter; hepatic enzymes; beta cells apoptosis; PPARs; AMPK; tyrosine kinase; and NF-κB pathways. As a result of this hypothesized combination, the over activation of these pathways may be prevented, while the needed action to improve diabetes may be achieved. At this time, these are no more than suggestions which need to be proven by research. To date, little is known about flavonoids to flavonoids interactions [235]. In addition, some flavonoids showed an opposite effect on the same pathway and both lead to the improvement of diabetes. For example, fisetin has an inhibitory effect, while morin has a stimulatory effect on glucose 6 phosphate dehydrogenase and the literature states that they both improve diabetes (Figure 1). Extensive studies are required to understand the reasons behind this action—is it because of different binding sites, bioavailability, tissue exposure, absorption, or circulating concentration of these compounds. A similar pattern with different flavonoids was observed with PPAR and NF-κB pathways (Table 1, Table 2, Table 3 and Table 4).

#### 3.1.3. Flavonoids and Metformin

Metformin is an oral anti-diabetic drug derived from the French lilac suitable for the treatment of diabetes with a well-known safety profile [236]. Comparing the effect of metformin and anthocyanins extracted from blueberries on blood glucose levels revealed that blueberry extract (595 mg/g total anthocyanins) led to a 33% to 51% reduction in blood glucose compared to 27% reduction seen with metformin [237]. Another study reported that quercetin stimulates an insulin dependent AMPK pathway, which is analogues to metformin activity [15]. In addition, a study which measured the effect of the co-treatment of metformin and flavone on breast cancer patients showed a significant inhibition in cell viability and an increase in apoptosis [238].

#### 3.1.4. Flavonoids for the Treatment of Cancer

Phytochemicals are successfully used in the treatment of various cancers by modulating apoptotic pathways through: Reactive oxygen species (ROS) elevation; DNA damage induction; and apoptotic protein activation as clearly discussed in Abotaleb et al., 2018 [50]. Although flavonoids target and improve both the intrinsic and extrinsic apoptotic protein in cancer, they solely target the intrinsic pathway (Figure 3). Quercetin has similar beneficial effects on cancer and diabetes by inhibiting PI3K pathway [239]. The administration of luteolin targeting the NF-κB pathway showed an inhibitory effect on cancer, while a stimulatory effect on diabetes [240].

### 3.2. Final Thoughts

Flavonoids, abundantly found in fruits and vegetables, have mostly beneficial effects on diabetes. Eating vegetables and fruits could help to lower blood sugar levels and to decrease the chance to develop diabetes. Generally, it is possible that their combination with other phytochemicals could enhance the anti-diabetic effects, but more research is needed to support this promising way to reduce blood sugar levels.

## Figures and Tables

**Table 1:** Representative flavonol and their underlying anti-diabetic effects.

Flavonoid Subclass	Name of Flavonoid	Structure of Flavonoid	Dietary Source	Metabolites Produced from Flavonoids	Function of Flavonoids	Mechanism of Action	Model Used		References
							In Vivo	In Vitro	
**Flavonol**	**1. Rutin**	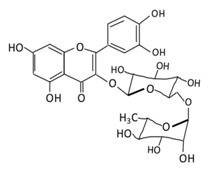	Oranges, grapes, limes, lemons, berries and peaches.	Metabolization depends on intestinal bacteria: (A) Bacillus 52 and Bacteroides 45 produce: Quercetin-3-*O*-glucoside and Leucocynaidin. (B) Bacteroides 42 and veillonella 32 produces: Leucocynaidin. (C) Bacteroides 22 hydrolysis produce: Quercetin-3-*O*-glucosie	(A) Antihyperglycmeic effect: (B) Hypolipemic effect	Inhibit α-glucosidase and α-amylase which reduce the absorption of glucose in small intestine Decrease G6Pase, PEPCK, glycogen phosphorylase, and fructose-1,6-bisphosphatase enzymes in liver and kidney Decrease the level of caspase 3 and increase the level of Bcl-2 which shows an anti-apoptotic activities Reduce the level of hemoglobin A1C (HbA1c) Activate the synthesis and translocation of GLUT4 that stimulate glucose transport to soleus muscle tissue Increase hexokinase activity in liver Improve the morphology of islets of Langerhans Reduce serum LDL, VLDL, triglyceride Inhibit lipid peroxidation Increase serum level of HDL Activate the expression of PPAR-γ which improve glucose uptake and insulin resistance	Streptozotocin induced diabetic rats Type 2 diabetic rat Streptozotocin induced diabetic wistar rats	Streptozotocin diabetic tissue	[81,83]
	**2. Fisetin**	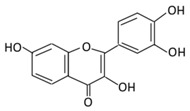	Onion, strawberries, and persimmon	(A) Glucuronide of fisetin (M1) (B) Glucuronide (M2) (C) Methoxylated metabolites of fisetin (M3)	(A) Antihyperglycmeic effect	Inhibit gluconeogenesis by inhibiting pyruvate transport into mitochondria Decrease glycogen breakdown which prevent hyperglycemia Reduce blood glucose, Hb1Ac, IL-1β, and NF-κB p65 unit Reduce the activity of glucose glucose-6-phosphate dehydrogenase activity	Streptozotocin induced diabetic rats		[107,108]
	**3. Kaempferol**	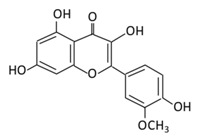	Cruciferous vegetables, tea, grapefruit, edible berries, and *Gingko biloba* L.	(A) Kaempferol-3-*O*-glucoside (B) Kaempferol-3-*O*-diglucoside	(A) Antihyperglycmeic effect: (B) Hypolipemic effect	Reduce serum glucose level and fasting blood glucose level Decrease the level of caspase 3 activity in β-cells Inhibit cellular apoptosis by improving anti-apoptotic Akt activities Improve cAMP signaling and insulin synthesis and secretion Improve glucose uptake by soleus muscles Reduce lipid peroxidation Decrease PPARγ expression through AMPK activity	Rats Streptozotocin (STZ)-induced diabetic rats High fat diet mice	Pancreatic β-cells	[90,91]
	**4. Quercetin**	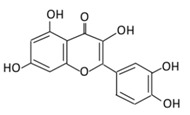	Black currants, cherries, apples and chokeberries	(A) Quercetin-3-*O*-glucoside (B) Quercetin -3-*O*-glucoside-7-*O*-glucoside (C) Quercetin-3-*O*-galactoside (D) Aglycone	(A) Antihyperglycmeic effect: (B) Hypolipemic effect	Inhibit insulin dependent activation of PI3K Inhibit GLUT2 which reduces the absorption of glucose in small intestine Block the activity of tyrosine kinase Improve GLUT4 translocation through the activation of AMPK Improve the recovery of cell proliferation Improve glucose absorption Reduce lipid peroxidation	Rats Streptozotocin (STZ)-induced diabetic rats High fat diet mice	Skeletal muscle cells Hepatocyte RINm5F β-cells	[68,69]
	**5. Isorhamnetin**	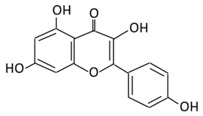	*Oenanthe javanica*, *Hippophae rhamnoides*, and *Ginkgo biloba L.*	(A) Isorhamnetin (B) isorhamnetin-3-*O*-galacto	(A) Antihyperglycmeic effect: (B) Hypolipemic effect:	Improve insulin secretion Increase glucose transporter 2 (GLUT2) Inhibit adipogenesis	HFD- induced C57BL/6 mice	3 T3-L1 cells	[102,103]
	**6. Morin**	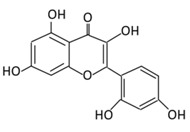	*Psidium guajava*, *Prunus dulcis* (Almond), *chlorophora tinctoria*, and fruits	(A) Morin glucuronides (B) Morin sulfates	(A) Antihyperglycmeic effect: (B) Hypolipemic effect.	Reduce hepatic NF-κB activation Reduce G6Pase and FDPase enzymatic activities Increase hexokinase and G6PD enzymatic activities Improve hyperglycemia, insulin resistance, and glucose intolerance Reduce lipid peroxidation Reduce hyperlipidemia Normalize the profile of lipid and lipoprotein	Streptozotocin (STZ)-induced diabetic rats High fructose fed rats HFD-STZ induced type 2 diabetic rats	Rats hepatocyte	[114,115]

**Table 2:** Representative flavanones and their underlying anti-diabetic effects.

Flavonoid Subclass	Name of Flavonoid	Structure of Flavonoid	Dietary Source	Metabolites Produced from Flavonoids	Function of Flavonoids	Mechanism of Action	Model Used		References
							In Vivo	In Vitro	
**Flavanones**	**7.Hesperidin**	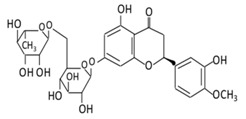	Orange citrus aurantium	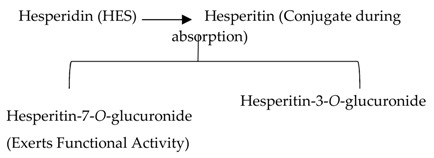	(A) Antihyperglycmeic effect: (B) Hypolipemic effect	Down- regulate the production of free radical and proinflammatory cytokines Reduce oxidative stress Reduce blood glucose level by affecting glucose enzymatic activities Increase glycogen concentration and hepatic glycolysis Reduce the level of TBARS which is a byproduct of lipid peroxidation Normalize adiponectin level Increase the activity of lactate dehydrogenase (LDH)	Alloxan-induceddiabetic rabbits Streptozotocin (STZ)-induced marginal type 1 diabetic rats (10g/kg diet)		[122,124]
	**8.Naringenin**	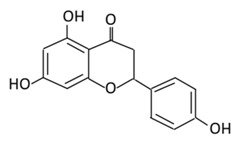	Grapefruit (*C. paradisi*), Chinese herbs like *C. aurantium*	Four forms could be present in the body two of them are major: (A) Naringenin glucuronides (Major form in serum) (B) Naringenin sulfates ( Major form in liver) (C) Free naringin (Not present in blood stream) D) Free naringenin (Not present in blood stream)	(A) Antihyperglycmeic effect: (B) Hypolipemic effect	Reduce poliprotein B secretion in the liver which mimic insulin effect Inhibit intestinal α-glucosidase activity which delays carbohydrates absorption Inhibit glucose uptake by inhibiting sodium glucose co-transporter Activate AMPK pathway which increase insulin sensitivity and glucose tolerance Reduce membrane lipid peroxidation Prevent apolipoprotein B overproduction and dyslipidemia Induce hypolipidemic activity	Streptozotocin (STZ)-induced diabetic rats High fat diet fed mice LDL receptor null mice Male Sprague-Dawley rats	INS-1E cells	[133,135]
	**9.Eriodictyol**	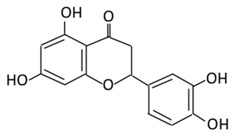	Lemon, Torr, *Eridictyon californicum*, *Millettia duchesnei De Wild*, and *Eupatorium arnottianum*	(A) Monoglucuronide M1 in the liver microsome (B) Monoglucuronide M2 in the liver microsome	(A) Antihyperglycmeic effect: (B) Hypolipemic effect:	Suppress oxidative stress Decrease Intercellular Adhesion Molecule 1 (ICAM-1), Vascular endothelial growth factor (VEGF), retinal TNFα, and Endothelial NOS (eNOS). Reactivate Akt phosphorylation Reduce lipid peroxidation Up-regulate the expression of PPARγ2 Up-regulate adipocyte- specific fatty acid binding protein	Streptozotocin induced diabetic rats (0.2%)	HepG2 cells Differentiated 3T3-L1 cells	[144,146]

**Table 3:** Representative flavones and their underlying anti-diabetic effects.

Flavonoid Subclass	Name of Flavonoid	Structure of Flavonoid	Dietary Source	Metabolites Produced from Flavonoids	Function of Flavonoids	Mechanism of Action	Model Used		References
							In Vivo	In Vitro	
**Flavones**	**10. Baicalein**	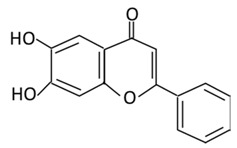	*Scutellaria lateriflora L*, and *Scutellaria baicalensis* Georgi	In Intestine: Baicalin will be converted into Baicalein and then absorbed rapidly. In the circulation: Baicalein will be converted to Baicalin	(A) Antihyperglycmeic effect: (B) Hypolipemic effect	Reduce the level of level of hemoglobin A1C (HbA1c) Suppress the activation of NF-κB Improve glucose tolerance and insulin secretion from pancreatic cells Improve viability of clonal β-cells which improves the production of NADH and NADPH Protect against β cells apoptosis Increase hexokinase activity in liver Activate MAPKs signaling pathway which reduce the effect of insulin resistance by phosphorylating Akt and IRS-1 and dephosphorylate NF-κB Suppress fatty acid synthesis	Obese diabetic mice Type 2 diabetic rats	CA1 hippocampal neurons	[187,190]
	**11. Luteolin**	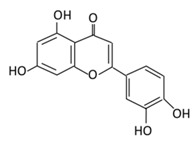	Parsley, broccoli, onoins leaves, celery, cabbages, apple skins, carrots, and peppers	Metabolization is medicated by UGTs and COMTs to produce: (A) Luteolin-7-glucuronide (Glucuronidated) (B) Luteolin-4-glucuronide (C) Chrysoeriol/diosmetic (Methylated) (D) Luteolin monoglucuronide (Major form in human serum	(A) Antihyperglycmeic effect: (B) Hypolipemic effect	Reduce cAMP response element binding protein and histone acetyl transferase activity of CBP/p300 (NF-κB coactivator) Reduce apoptosis Up-regulate the espression of synaptic protein which target brain cells Improve insulin secretion by supressing Maf A through NF-κB signiling pathway Activate PPAR-γ which targets adiponectin, leptin and GLUT4 genes	Obese mice Streptozotocin induced diabetic rats Diabetic rats	Endothelium cells Human monocytes cells	[155,157]
	**12. Diosmin**	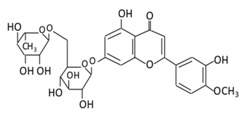	Citrus fruites, and *Scrophularia nodosa L.*	(A) Diosmin (Not excreted in urine) (B) Diosmetin (Not excreted in urine) (C) Minor metabolites in the form of glucuronic acid conjugate (Excreted in urine)	(A) Antihyperglycmeic effect: (B) Hypolipemic effect:	Reduce the level of hemoglobin A1C (HbA1c) due to increase in glutathione peroxidase (GPx) Decrease G6Pase, PEPCK, and fructose-1,6-bisphosphatase enzymes Reduce plasma glucose and increase plasma insulin by activating anti-oxidant enzymes Reduce hyperglycemia by inducing β-endorphin Increase hexokinase and glucose-6-phosphate dehydrogenase activity Reduce lipid peroxidation	Streptozotocin nicotinamide induced diabetic rats		[179,180]
	**13. Apigenin**	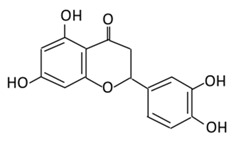	Onion, oranges, tea, parsley, chamomile, *Hypericum perforatum L*, wheat sprouts	Metabolization occurs through two phases: Phase (1): Apigenin produce three monohydroxylated: a) Luteolin b) Scutellarien c) iso-scutellarein Phase(2): Luteolin produce: a) Four monoglucuroconjugates b) Two Sulfoconjugate c) One methyl conjugate	(A) Antihyperglycmeic effect: (B) Hypolipemic effect:	Reduce cellular antioxidants Attenuate cell damage in pancreatic β-cells Improve the morphology of the cells Improve GLUT4 translocation which lowers glucose level Increase serum cholesterol Increase lipid peroxidation	Streptozotocin induced diabetic rats (0.2%)	HepG2 cells Differentiated3T3-L1 cells	[147,149]
	**14.Tangeretin**	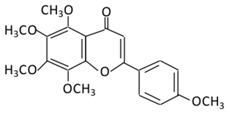	*Poncirus trifoliate L,* citrus fruit rinds, and mandarin orange	Metabolization is medicated by CYP1A1 and CYP1A2 to produce: (A) 4′ hydroxy - 5, 6, 7, 8 tetramethoxyflavone (4′-OH-TMF)	(A) Antihyperglycmeic effect: (B) Hypolipemic effect:	Reduce blood glucose and HbA1c level Reduce the secretion of insulin resistance factor Increase the secretion level of insulin and insulin sensitizing factor Enhances glycolytic enzyme in the liver Reduce total cholesterol and adipocytokines level	Rats Streptozotocin (STZ)-induced diabetic rats High fat diet mice	Pancreatic β-cells	[160,162]
	**15. Wogonin**	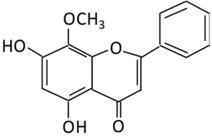	*Scutellaria baicalensis* Georgi	(A) Wogonin-7-beta-D-glucuronide (Major metabolites) (B) Wogonin-5-beta-D-glucuronide	(A) Antihyperglycmeic effect: (B) Hypolipemic effect:	Reduce hyperglycemia and lipid droplets accumulation in the liver Increase vascular permeability and the expression of cell adhesion molecules Activate NF-κB and AMPK pathways Activate PPARα which has a beneficial effect on lipid metabolism	db/db mice	3T3-L1 cells	[173,175]
	**16. Chrysin**	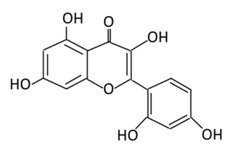	*passiflora caerulea (L,),* honey, *Tilia tomentosa* Moench, and *Pelargonium crispum (Berg.)*	(A) Chrysin glucuronides (M1) (B) Chrysin sulfates (M2)	(A) Antihyperglycmeic effect: (B) Hypolipemic effect:	Reduce the level of pro-inflammatory cytokines that helps in the prevention of diabetic neuropathy Reduce blood glucose Improve renal pathology with the suppression of TGF-β, collagen-IV, and fibronectin Improve insulin level Reduce lipid peroxidation		INS-1E cells	[167,169]

**Table 4:** Representative isoflavones, anthocyanins and their underlying anti-diabetic effects.

Flavonoid Subclass	Name of Flavonoid	Structure of Flavonoid	Dietary Source	Metabolites Produced from Flavonoids	Function of Flavonoids	Mechanism of Action	Model used		References
							In Vivo	In Vitro	
**Isoflavones**	**17. Genistein**	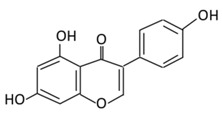	Soybeans, kudzu, and fava bean	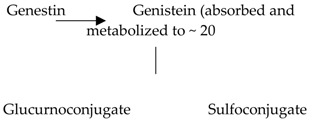	(A) Antihyperglycmeic effect: (B) Hypolipemic effect	Reduce hyperglycemia through the activity of cAMP/ PKA pathway Decrease Intercellular Adhesion Molecule 1 (ICAM-1) and p-ERK Inhibit the activity of tyrosine kinase Improve glucose intolerance and β-cells mass Decrease urinary excretion of TBARs	Streptozotocin (STZ)-induced diabetic rats Obese diabetic mice Nongenetictype 2 diabetic mice	INS-1 cells Human islet β-cells	[195,199]
	**18. Daidzein**	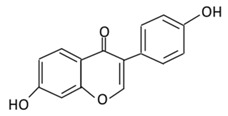	Soybeans, nuts, and soy milk	(A) Daidzin	(A) Antihyperglycmeic effect:	Decrease blood glucose, total cholesterol, and AMPK phosphorylation	Golden Syrian hamsters		[202,204]
**Anthocyanins**	**19. Cyanidin**	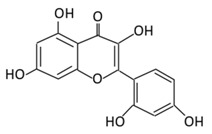	Bilberry, blueberry, grapes, blackberries, hawthorn, acai berry, and raspberry	(A) Anthocyanidin glucuronide conjugates (Major form in urine) (B) Simple Aglycones (Second major in urine) (C) Anthocyanidin methyl glucuronide conjugates (8 forms) (D) Cyanidin-3-glucoside E) Cyanidin-3-galactoside	(A) Antihyperglycmeic effect: (B) Hypolipemic effect:	Inhibit α-glucosidase and α-amylase which reduce the absorption of glucose in small intestine Reduce fasting glucose level Prevent pancreatic apoptosis Improve antioxidant status which protects hepatocytes from HG-induced damage Attenuate aortic lipid peroxidation	Streptozotocin (STZ)-induced diabetic rats db/db rats high fat diet fed mice	Mouse hepatocyte	[207,209]
	**20.Delphinidin**	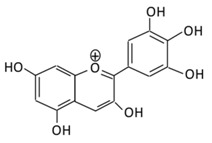	Dark grapes, eggplant, berries, red cabbage, carrot, and tomato	(A) 4′-*O*- methyl delphinidin 3-*O*-beta-d- glucopyranoside	(A) Antihyperglycmeic effect:	Reduce the glycation rate of HbA1c Prevents diabetes associated injuries such as endothelial cell function	Diabetic mouse		[213,215]
	**21.Pelargonidin**	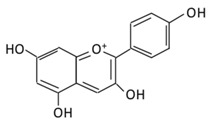	Bilberry and *ficus bengalensis* Linn	(A) Pelargonidin-*O*-glucuronide (B) Pelargonidin-3-galactoside	(A) Antihyperglycmeic effect: (B) Hypolipemic effect:	Reduce hyperglycemia Reduce the level of antioxidant defensive enzymes Stimulate insulin secretion Reduce the level of TBARS which is a byproduct of lipid peroxidation	Streptozotocin (STZ)-induced diabetic rats Diabetic rats		[217,219]

## Abbreviations

DM	diabetes mellitus
GLUT	glucose transporter
HMIT	myo-inositol transporter
IRS	insulin resistance substrate
IR	insulin receptor
PTP	protein tyrosine phosphatase
PTEN	tensin homologue
FFAs	free fatty acids
TNF	tumor necrosis factor
SOCS	suppressor of cytokine signaling
IAPP	islet amyloid polypeptide
ER	endoplasmic reticulum
PPAR	peroxisome proliferator activated receptor
AMPK	adenosine monophosphate activated protein kinase
PI3K	Phosphoinositide 3-kinases
STZ	streptozotocin
NF-κB	nuclear Factor kappa-light-chain-enhancer of activated B cells
Bcl-2	B-cell lymphoma 2
HbA1c	hemoglobin A1C (glycated hemoglobin)
NO	nitric oxide
G6PD	glucose-6-phosphate dehydrogenase
G6Pase	glucose 6-phosphatase
HOMA-IR	homeostatic model assessment of insulin resistance
FDPase	fructose-1,6-diphosphatase
Msft	micro-autologous fat transplantation
IL-6	Interleukin 6
DN	diabetic neuropathy
GPx	glutathione peroxidase
HFD	high fat diet
AGEs	Advanced glycation end product
PKA	protein kinase A
NOD	non-obese diabetic
C3G	cyanidin-3-glucoside
HG	high glucose
TBARS	thiobarbituric acid reactive substances
ROS	reactive oxygen species
TC	total cholesterol
LDL	light density lipoprotein
TG	triglyceride
CAT	computerized axial tomography
MDA	muscular dystrophy association
RPTEC	renal proximal tubule epithelial cells
NRK-52E	rat kidney epithelial cells
